# Supplementary material for: Analysis of PPARγ Signaling Activity in Psoriasis
Source: Int J Mol Sci. 2021 Aug 10;22(16):8603. doi: 10.3390/ijms22168603 (PMC8395241; doi:10.3390/ijms22168603)
Supplement: Supplementary file 1 [file ijms-22-08603-s001.zip › Supplemental materials_Analysis of PPARg signaling activity in psoriasis/Pathway models/Models images and html files/Model of PPARG related pathways in psoriasis (short version)/7477.html]

CXCL9


# Protein CXCL9

|  |  |
| --- | --- |
| URN | urn:agi-llid:4283 |
| Total Entities | 0 |
| Connectivity | 2028 |
| Name | CXCL9 |
| Description | chemokine (C-X-C motif) ligand 9 |
| Notes | This antimicrobial gene encodes a protein thought to be involved in T cell trafficking. The encoded protein binds to C-X-C motif chemokine 3 and is a chemoattractant for lymphocytes but not for neutrophils. [provided by RefSeq, Sep 2014] |
| Primary Cell Localization | Extracellular |
| Class | Ligand |

---

|  |  |
| --- | --- |
| Pathway | CXC Chemokine Receptor Signaling |
|  | Macrophage M1 Lineage |
|  | Nociception Expression Targets Signaling |
|  | CCR1 Expression Targets |
|  | Proteins Involved in Atherosclerosis |
|  | Proteins Involved in Psoriasis |
|  | Toll-like Receptors in beta-Cell |
|  | Hashimoto's Thyroiditis |
|  | B-Cell Chronic Lymphocytic Leukemia |
|  | IL6/IL12 Signaling Activates Immune System in Multiple Sclerosis |
|  | Proteins Involved in Glomerulonephritis |
|  | CD8 -> STAT Expression Targets |
|  | EGFR -> Expression Targets in Skin |
|  | IFNA1/IFNR Expression Targets |
|  | IFNG/IFNR Expression Targets |
|  | GPCRs Family -> Expression Targets in Lymphoid System and Blood |
|  | Interleukin Receptors -> Expression Targets in Lymphoid System and Blood |
|  | Chemokine Receptor Family -> Expression Targets in Lymphoid System |
|  | PRL/PRLR Expression Targets |
|  | PRL/GHR -> STAT Expression Targets |
|  | PRL/GHR -> NF/kB/ELK/SRF/MYC Expression Targets |
|  | TNF -> NF-kB Expression Targets |
|  | CD40LG -> NF-kB/ELK/SRF -> CREB/NFATC Expression Targets |
|  | TNF -> STAT Expression Targets |
|  | TLR4 -> NF-kB/IRF Expression Targets |
|  | pprarg neg, uknown targets, ps-positive |
|  | prarg neg,ukn expres targets, ps-positive |
|  | PPARG negative targets |
|  | pparg expr target\_regulators |
|  | Neighbors of disease exacerbation |
|  | Neighbors of tumor progression |
|  | Neighbors of cancer progression |
|  | Neighbors of overall survival |
|  | Neighbors of disease severity |
|  | Neighbors of tumor size |
|  | Neighbors of mortality |
|  | Neighbors of survival time |
|  | Neighbors of graft survival |
|  | Neighbors of lethality |
|  | Neighbors of tumor regression |
|  | Neighbors of tumor response |
|  | Neighbors of long-term survival |
|  | Neighbors of treatment outcome |
|  | Neighbors of fibrosis progression |
|  | Neighbors of cell density |
|  | Neighbors of viral load |
|  | Neighbors of T-cell count |
|  | Neighbors of disease exacerbation |
|  | Neighbors of tumor progression |
|  | Neighbors of cancer progression |
|  | Neighbors of overall survival |
|  | Neighbors of disease severity |
|  | Neighbors of tumor size |
|  | Neighbors of mortality |
|  | Neighbors of survival time |
|  | Neighbors of graft survival |
|  | Neighbors of lethality |
|  | Neighbors of tumor regression |
|  | Neighbors of tumor response |
|  | Neighbors of long-term survival |
|  | Neighbors of treatment outcome |
|  | Neighbors of fibrosis progression |
|  | Neighbors of cell density |
|  | Neighbors of viral load |
|  | Neighbors of T-cell count |
|  | Neighbors of cell development |
|  | Neighbors of immune response |
|  | Neighbors of cell population |
|  | Neighbors of T-cell development |
|  | Neighbors of inflammatory response |
|  | Neighbors of wound healing |
|  | Neighbors of angiogenesis |
|  | Neighbors of innate immune response |
|  | Neighbors of cell function |
|  | Neighbors of cell formation |
|  | Neighbors of adaptive immune response |
|  | Neighbors of epithelial to mesenchymal transition |
|  | Neighbors of immunity |
|  | Neighbors of chemotaxis |
|  | Neighbors of dendritic cell differentiation |
|  | Neighbors of cell survival |
|  | Neighbors of T-cell proliferation |
|  | Neighbors of tissue remodeling |
|  | Neighbors of cell adhesion |
|  | Neighbors of T-cell activation |
|  | Neighbors of endothelial cell proliferation |
|  | Neighbors of fibrogenesis |
|  | Neighbors of tumor growth |
|  | Neighbors of dendritic cell development |
|  | Neighbors of tissue repair |
|  | Neighbors of cell count |
|  | Neighbors of cell infiltration |
|  | Neighbors of cell interaction |
|  | Neighbors of endothelial cell migration |
|  | Neighbors of lipid storage |
|  | Neighbors of pregnancy |
|  | Neighbors of cell motility |
|  | Neighbors of bone resorption |
|  | Neighbors of monocyte migration |
|  | Neighbors of fibroblast proliferation |
|  | Neighbors of regeneration |
|  | Neighbors of cell migration |
|  | Neighbors of cancer growth |
|  | Neighbors of transendothelial migration |
|  | Neighbors of phagocytosis |
|  | Neighbors of immune system activation |
|  | Neighbors of leukocyte migration |
|  | Neighbors of leukocyte recruitment |
|  | Neighbors of monocyte recruitment |
|  | Neighbors of cell invasion |
|  | Neighbors of ossification |
|  | Neighbors of T-cell function |
|  | Neighbors of immune system function |
|  | Neighbors of T-cell response |
|  | Neighbors of tumor immunity |
|  | Neighbors of cell growth |
|  | Neighbors of ROS generation |
|  | Neighbors of first trimester pregnancy |
|  | Neighbors of sensitization |
|  | Neighbors of hemato-encephalic barrier |
|  | Neighbors of cellular immune response |
|  | Neighbors of colony formation |
|  | Neighbors of cell differentiation |
|  | Neighbors of stem cell migration |
|  | Neighbors of psoriasis |
|  | Neighbors of inflammatory disease |
|  | Neighbors of rheumatoid arthritis |
|  | Neighbors of atherosclerosis |
|  | Neighbors of fibrosis |
|  | Neighbors of colitis |
|  | Neighbors of arthritis |
|  | Neighbors of inflammatory bowel disease |
|  | Neighbors of injury |
|  | Neighbors of vascular remodeling |
|  | Neighbors of metastasis |
|  | Neighbors of carcinogenesis |
|  | Neighbors of multiple sclerosis |
|  | Neighbors of chronic inflammation |
|  | Neighbors of inflammation |
|  | Neighbors of tumor microenvironment |
|  | Neighbors of pathological angiogenesis |
|  | Neighbors of diabetes mellitus |
|  | Neighbors of asthma |
|  | Neighbors of pneumonia |
|  | Neighbors of liver fibrosis |
|  | Neighbors of myocardial infarction |
|  | Neighbors of leukocyte infiltration |
|  | Neighbors of airway inflammation |
|  | Neighbors of hepatitis |
|  | Neighbors of infection |
|  | Neighbors of vascular injury |
|  | Neighbors of macrophage infiltration |
|  | Neighbors of liver injury |
|  | Neighbors of neuroinflammation |
|  | Neighbors of death |
|  | Neighbors of autoimmune disease |
|  | Neighbors of dermatitis |
|  | Neighbors of heart disease |
|  | Neighbors of diabetic nephropathy |
|  | Neighbors of lung injury |
|  | Neighbors of immunopathology |
|  | Neighbors of hepatocellular carcinoma |
|  | Neighbors of colorectal cancer |
|  | Neighbors of cardiac remodeling |
|  | Neighbors of breast cancer |
|  | Neighbors of systemic lupus erythematosus |
|  | Neighbors of melanoma |
|  | Neighbors of neoplasm invasion |
|  | Neighbors of kidney disease |
|  | Neighbors of heart failure |
|  | Neighbors of stroke |
|  | Neighbors of cancer |
|  | Neighbors of neoplasm |
|  | Neighbors of vasculitis |
|  | Neighbors of respiratory hypersensitivity |
|  | Neighbors of synovitis |
|  | Neighbors of liver disease |
|  | Neighbors of prostate cancer |
|  | Neighbors of osteolysis |
|  | Neighbors of ovarian cancer |
|  | Neighbors of hypertension |
|  | Neighbors of allograft rejection |
|  | Neighbors of epithelial cell |
|  | Neighbors of keratinocyte |
|  | Neighbors of vascular smooth muscle cell |
|  | Neighbors of umbilical vein endothelial cell |
|  | Neighbors of monocyte |
|  | Neighbors of alveolar macrophage |
|  | Neighbors of synoviocyte |
|  | Neighbors of macrophage |
|  | Neighbors of activated monocyte |
|  | Neighbors of fibroblast |
|  | Neighbors of airway epithelial cell |
|  | Neighbors of PBMC |
|  | Neighbors of stromal cell |
|  | Neighbors of osteoblast |
|  | Neighbors of pneumocyte |
|  | Neighbors of endometrium cell |
|  | Neighbors of microglia |
|  | Neighbors of intestine epithelium cell |
|  | Neighbors of mononuclear cell |
|  | Neighbors of hepatic stellate cell |
|  | Neighbors of hepatocyte |
|  | Neighbors of decidual cell |
|  | Neighbors of kidney mesangial cell |
|  | Neighbors of endothelial cell |
|  | Neighbors of dendritic cell |
|  | Neighbors of Kuppfer cell |
|  | Neighbors of inflammatory cell |
|  | Neighbors of bone marrow macrophage |
|  | Neighbors of osteoclast |
|  | Neighbors of neutrophil |
|  | Neighbors of astrocyte |
|  | Neighbors of activated T-cell |
|  | Neighbors of capillary endothelial cell |
|  | Neighbors of immunocompetent cell |
|  | Neighbors of granulocyte |
|  | Neighbors of cancer-associated fibroblast |
|  | Neighbors of myeloid cell |
|  | Neighbors of mesenchymal stroma cell |
|  | Neighbors of stem cell |
|  | Neighbors of tumor infiltrating macrophage |
|  | Neighbors of sinusoidal cell |
|  | Neighbors of B-cell |
|  | Neighbors of leukocyte |
|  | Neighbors of insulin-secreting cell |
|  | Neighbors of T-cell |
|  | Neighbors of M1 macrophage |
|  | Neighbors of myofibroblast |
|  | Neighbors of mast cell |
|  | Neighbors of spleen cell |
|  | Neighbors of neuroglia |
|  | Neighbors of thyroid epithelial cell |
|  | Neighbors of podocyte |
|  | Neighbors of conventional dendritic cell |
|  | Neighbors of pericyte |
|  | Neighbors of epidermis cell |
|  | Neighbors of lymphocyte |
|  | Neighbors of Langerhans cell |
|  | Neighbors of thymic epithelial cell |
|  | Neighbors of lymphoid cell |
|  | Neighbors of activated B-cell |
|  | Neighbors of liver |
|  | Neighbors of kidney |
|  | Neighbors of skin |
|  | Neighbors of intestine |
|  | Neighbors of lymph node |
|  | Neighbors of heart |
|  | Neighbors of gastrointestinal tract |
|  | Neighbors of microvessel |
|  | Neighbors of placenta |
|  | Neighbors of synovial membrane |
|  | Neighbors of uterus |
|  | Neighbors of islets of Langerhans |
|  | Neighbors of left ventricle |
|  | Neighbors of glands |
|  | Neighbors of eye |
|  | Neighbors of NF-kB family |
|  | Neighbors of oxidized LDL |
|  | Neighbors of IL1 family |
|  | Neighbors of PPAR |
|  | Neighbors of Jun/Fos |
|  | Neighbors of PI3K |
|  | Neighbors of mitogen-activated protein kinase |
|  | Neighbors of IFNAR ligand |
|  | Neighbors of TLR |
|  | Neighbors of cytokine |
|  | Neighbors of interferon |
|  | Neighbors of JAK |
|  | Neighbors of ovalbumin |
|  | Neighbors of inflammatory cytokine |
|  | Neighbors of IL23 |
|  | Neighbors of T-cell receptor |
|  | Neighbors of IL12 |
|  | Neighbors of prostaglandin-endoperoxide synthase |
|  | Neighbors of aldehyde reductase |
|  | Neighbors of non-coding RNA |
|  | Neighbors of histone H3 |
|  | Neighbors of STAT5 |
|  | Neighbors of TORC1 |
|  | Neighbors of phosphodiesterase |
|  | Model of PPARG signaling in psoriasis |
|  | PPARG negative regulators and targets |
|  | Model of PPARG related pathways in psoriasis (short version) |
|  | 4\_1\_Polymorphisms associated with inflammatory bowel diseases |

---

|  |  |
| --- | --- |
| MedScan ID | 4283 |

---

|  |  |
| --- | --- |
| LocusLink ID | 4283 |

---

|  |  |
| --- | --- |
| Alias | CXC chemokine ligand 9 |
|  | Gamma interferon induced monokine |
|  | monokine induced by IFN-gamma |
|  | Small inducible cytokine B9 |
|  | chemokine CXC ligand 9 |
|  | C-X-C motif chemokine ligand 9 |
|  | small inducible cytokine b subfamily (cys-x-cys), member 9 |
|  | MIG protein |
|  | chemokine (C-X-C motif) ligand 9 |
|  | chemokine CXCL9 |
|  | Humig |
|  | SZ09 |
|  | small inducible cytokine b subfamily (cys-x-cys), member IX |
|  | CXC ligand 9 |
|  | monokine induced by gamma interferon |
|  | MGC105312 |
|  | C-X-C motif chemokine 9 |
|  | MIG |
|  | CXCL9 chemokine |
|  | crg-10 |
|  | CXCL9 |
|  | protein MIG |
|  | monokine induced by interferon gamma |
|  | crg X |
|  | M119 |
|  | SZ09\_HUMAN |
|  | interferon gamma-induced HuMIG |
|  | SCYB9 |
|  | monokine induced by IFNG |
|  | CMK |
|  | gamma-interferon-induced monokine |
|  | monokine induced by interferon-gamma |
|  | small-inducible cytokine B9 |

---

|  |  |
| --- | --- |
| GO ID | 0048248 |
|  | 0008009 |
|  | 0005125 |
|  | 0007186 |
|  | 0007189 |
|  | 0061844 |
|  | 0007267 |
|  | 0006968 |
|  | 0071222 |
|  | 0070098 |
|  | 0006935 |
|  | 0006952 |
|  | 0051607 |
|  | 0006955 |
|  | 0006954 |
|  | 0030595 |
|  | 0030593 |
|  | 0045663 |
|  | 1901741 |
|  | 0051281 |
|  | 0042127 |
|  | 0007165 |
|  | 0009897 |
|  | 0005576 |
|  | 0005615 |
|  | 0060326 |
|  | 0030816 |
|  | 0043950 |
|  | 0002690 |
|  | 0032496 |
|  | 0010818 |

---

|  |  |
| --- | --- |
| KEGG ID | hsa:4283 |

---

|  |  |
| --- | --- |
| Organism | Homo sapiens {Organism urn:agi-taxid:9606} |
|  | Homo sapiens |

---

|  |  |
| --- | --- |
| OMIM ID | 601704 |

---

|  |  |
| --- | --- |
| Hugo ID | 7098 |
|  | HGNC:7098 |

---

|  |  |
| --- | --- |
| Human chromosome position | 4q21.1 |
|  | 4q21 |

---

|  |  |
| --- | --- |
| Swiss-Prot Accession | Q07325 |
|  | Q07325.1 |
|  | Q503B4 |

---

|  |  |
| --- | --- |
| PIR ID | JN0470 |

---

|  |  |
| --- | --- |
| GenBank ID | NC\_000004 |
|  | NM\_002416 |
|  | NP\_002407 |
|  | AC112719 |
|  | CH471057 |
|  | EAX05763 |
|  | EAX05764 |
|  | AK311852 |
|  | BAG34794 |
|  | BC025745 |
|  | BC042178 |
|  | BC063122 |
|  | AAH63122 |
|  | BC095396 |
|  | AAH95396 |
|  | DB141449 |
|  | X72755 |
|  | CAA51284 |
|  | Q07325 |
|  | NC\_018915 |
|  | AMYH02009503 |
|  | HQ447615 |
|  | ADQ32101 |
|  | AC\_000136 |
|  | ABBA01063355 |

---

|  |  |
| --- | --- |
| Swiss-Prot ID | CXCL9\_HUMAN |

---

|  |  |
| --- | --- |
| Cell Localization | Secreted |

---

|  |  |
| --- | --- |
| Ensembl ID | ENSG00000138755 |
|  | ENSP00000354901.4 |
|  | ENST00000264888.5 |
|  | ENSP00000354901 |
|  | ENST00000264888 |

---

|  |  |
| --- | --- |
| Unigene ID | Hs.77367 |

---

|  |  |
| --- | --- |
| Shape | Rhomb |

---
